# Modeling characterization of the vertical and temporal variability of environmental DNA in the mesopelagic ocean

**DOI:** 10.1038/s41598-021-00288-5

**Published:** 2021-10-28

**Authors:** Elizabeth Andruszkiewicz Allan, Michelle H. DiBenedetto, Andone C. Lavery, Annette F. Govindarajan, Weifeng G. Zhang

**Affiliations:** 1 Department of Applied Ocean Physics and Engineering, Woods Hole Oceanographic Institution, Woods Hole, MA USA; 2 Department of Physical Oceanography, Woods Hole Oceanographic Institution, Woods Hole, MA USA; 3 Department of Biology, Woods Hole Oceanographic Institution, Woods Hole, MA USA; 4 Present Address: School of Marine and Environmental Affairs, University of Washington, Seattle, WA USA; 5 Present Address: Department of Mechanical Engineering, University of Washington, Seattle, WA USA

**Keywords:** Ecological genetics, Ocean sciences, Physical oceanography, Molecular ecology

## Abstract

Increasingly, researchers are using innovative methods to census marine life, including identification of environmental DNA (eDNA) left behind by organisms in the water column. However, little is understood about how eDNA is distributed in the ocean, given that organisms are mobile and that physical and biological processes can transport eDNA after release from a host. Particularly in the vast mesopelagic ocean where many species vertically migrate hundreds of meters diurnally, it is important to link the location at which eDNA was shed by a host organism to the location at which eDNA was collected in a water sample. Here, we present a one-dimensional mechanistic model to simulate the eDNA vertical distribution after its release and to compare the impact of key biological and physical parameters on the eDNA vertical and temporal distribution. The modeled vertical eDNA profiles allow us to quantify spatial and temporal variability in eDNA concentration and to identify the most important parameters to consider when interpreting eDNA signals. We find that the vertical displacement by advection, dispersion, and settling has limited influence on the eDNA distribution, and the depth at which eDNA is found is generally within tens of meters of the depth at which the eDNA was originally shed from the organism. Thus, using information about representative vertical migration patterns, eDNA concentration variability can be used to answer ecological questions about migrating organisms such as what depths species can be found in the daytime and nighttime and what percentage of individuals within a species diurnally migrate. These findings are critical both to advance the understanding of the vertical distribution of eDNA in the water column and to link eDNA detection to organism presence in the mesopelagic ocean as well as other aquatic environments.

## Introduction

The ocean’s mesopelagic zone is 200 to 1000 m deep and represents the largest habitat on earth, supporting considerable biomass and biodiversity^1^. However, it remains poorly studied, mainly due to its massive size and inaccessibility^2^. Researchers are developing new tools to investigate this ecosystem, especially as the importance of the habitat and its inhabitants roles in the global carbon cycle and food webs become better known^3^. Additionally, there is increasing interest in exploitation of mesopelagic biomass for nutraceutical and pharmaceutical purposes^3^. However, little is known about mesopelagic organisms, including information about food webs, life histories and behavior, and biomass and biodiversity^3^. Current methods utilized to explore mesopelagic biodiversity include video surveys by tethered or un-tethered autonomous underwater vehicles (AUVs)^4^ or remotely operated vehicles (ROVs)^5^, net tows or midwater trawls to capture organisms^6^, and acoustic surveys to infer biomass from shipboard or in-situ systems^7^. Though these methods provide important and valuable information, they each have limitations, including avoidance behavior of organisms and lack of taxonomic resolution either by camera images or inferring biomass by sound scattering^8–10^. Alongside traditional methods, new and innovative methods are being developed to answer questions about the mesopelagic such as: how is biomass distributed, what factors affect that distribution, and what taxa are primarily responsible for the biological removal of carbon from the surface due to diel vertical migration?^11–14^.

A promising alternative for studying mesopelagic and other marine organisms is the analysis of environmental DNA (eDNA) captured from water samples^15–19^. Relatively small volumes of water (mL to L) can provide information about the presence of many species from microbes to mammals through the use of molecular tools such as quantitative PCR (qPCR) or high-throughput sequencing (often referred to as metabarcoding)^20,21^. Benefits of using eDNA for biomass surveys include the relative ease of collecting water rather than organisms and the ability to detect taxa that are rare or traditionally hard to sample with existing methods such as fragile, gelatinous organisms^19,22^. Recent work has investigated applying eDNA methods to conduct biomass and biodiversity surveys in marine and freshwater environments, often comparing censuses of aquatic organisms by eDNA methods to traditional methods, recognizing that each data set provides different information^11,23–25^. Most studies find that eDNA and traditional methods detect many of the same taxa, yet each method also detects taxa that are overlooked by the other method. This can be due to known biases in collection methods (e.g., size selection of organisms by net mesh size or biases in environmental DNA sample processing such as inefficient PCR amplification or incomplete reference sequence databases^11^), but also can be explained by the inherent difference in surveying taxa versus surveying eDNA shed by taxa. Thus, there remain great uncertainties in how to link observed eDNA signals with organism presence and biomass as several questions remain about the source, fate, and transport of eDNA and how these processes affect eDNA data interpretation^26^.

Inferring the presence of taxa by eDNA in a water sample relies on opportunistically sampling any residual material shed from an organism. While this circumvents certain biases of traditional methods such as fish avoiding nets or size-selecting fish with a given mesh size, it also introduces uncertainty in the spatial and temporal resolution of the eDNA signal (i.e., how long ago and how far away did the eDNA leave the organism). Determining the relevant time and length scales of eDNA signals requires consideration of many complicated and inter-dependent processes, including how much and what forms of eDNA are shed by an organism, eDNA persistence and transport, and technical considerations of eDNA sampling and laboratory and bioinformatic processing^27^. Notably, these processes are also all time-varying and difficult to isolate to study independently.

Previous work suggests that eDNA comes in a variety of forms ranging from intra-cellular to extra-cellular, and that the form of eDNA can change over time (e.g., large particles break down into small particles) and will impact persistence times and transport properties^26,28–30^. Persistence, or decay, of eDNA is also expected to be determined by both biotic and abiotic factors, including but not limited to water temperature, pH, dissolved oxygen, nutrient concentrations, and abundance and composition of microbial communities (see Collins et al., 2018^31^ and Andruszkiewicz Allan et al., 2020^29^ for reviews). Finally, whether dissolved or particulate, eDNA is expected to be transported via physical processes including advection, dispersion, settling, and resuspension near benthic habitats^27,32,33^. Studies have demonstrated distinct differences in communities as inferred by eDNA on relatively small spatial scales in observational data^17,34,35^ and have paired observations with transport models in riverine^36,37^ and coastal/nearshore marine environments^32,38,39^. Despite the efforts to understand these complicated processes and all their interactions, it remains difficult to identify the time and location a measured eDNA signal was shed by the host organism. Furthermore, the processes described above focus on the fate and transport of eDNA after it has been shed from an organism and do not consider the movement of the host organism. In the ocean, organisms are moving in space and time while shedding eDNA. Little is understood about how eDNA from moving point sources (i.e., organisms) relate to eDNA concentrations measured in water samples.

In the mesopelagic ocean, many organisms exhibit diel vertical migration (DVM), residing at deeper depths in the daytime and migrating up to the surface at night to feed^1,40,41^. Because the timescale of eDNA persistence^31^ is generally of the same order of magnitude as the timescale of daily migrations, the resulting eDNA concentration profile is likely sensitive to the vertical migration characteristics. The eDNA variability in the *vertical* direction is thus of first-order importance. Of specific interest is the connection of the temporal and vertical variability of the eDNA concentration with the migration behavior.

**Figure 1:**
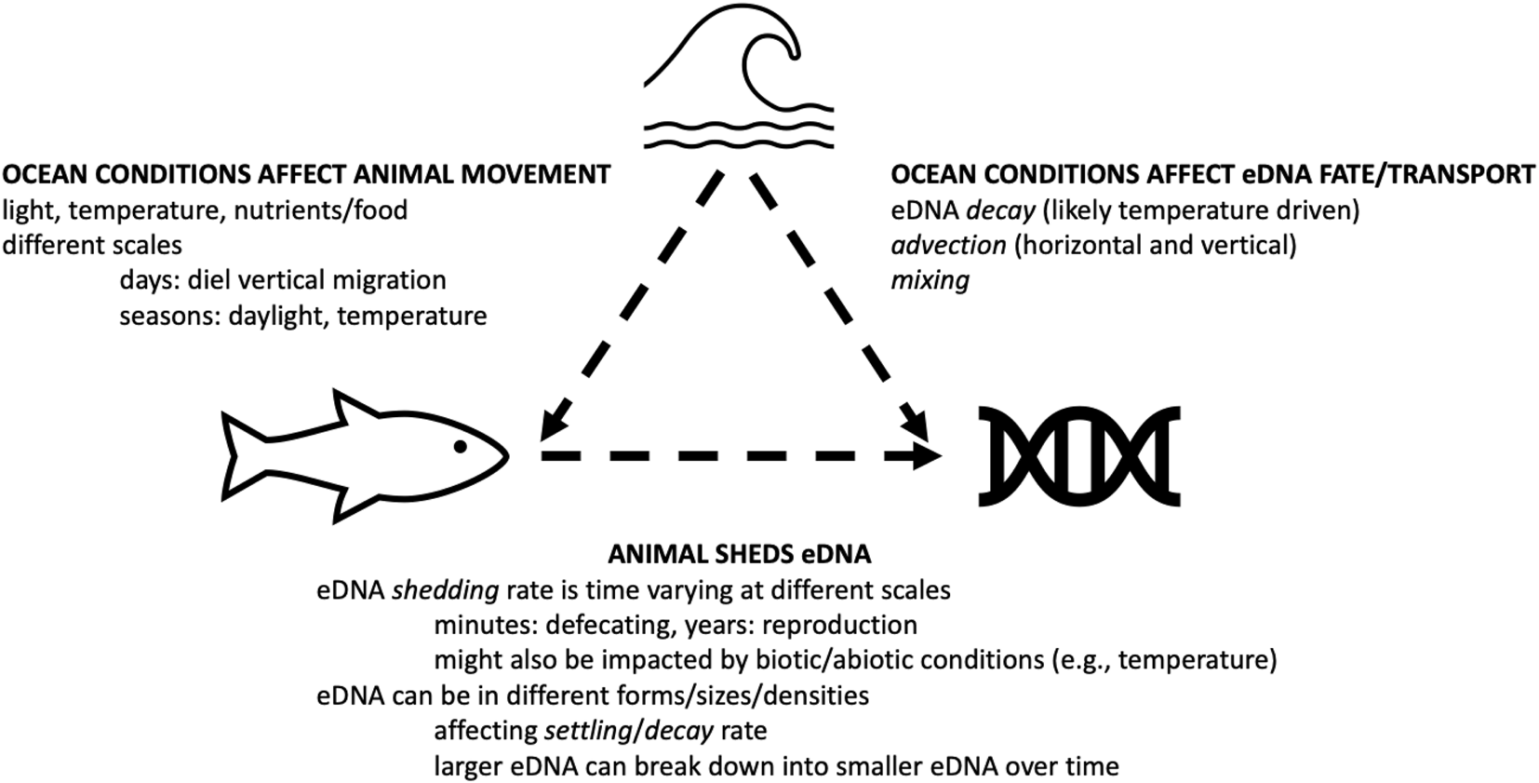
Conceptual model of the contributions of various factors to the eDNA vertical profiles. Organisms move in space and time while continuously shedding eDNA into the water column. After eDNA is shed from the organisms, it is subject to the influence of various transport, mixing and decaying processes, which also impact the vertical distribution of eDNA in the water column.

Here, we use a mechanistic model to generate vertical profiles of eDNA concentrations that are affected by organism behaviors (e.g., DVM), eDNA processes (e.g., decay, breakdown, and settling), and physical transport (e.g., mixing and advection) (Fig. 1). Our approach allows for an investigation of the vertical depth range of eDNA signals by separating the movement of the organism itself and the movement of the eDNA. The mechanistic model also demonstrates the sensitivity of the eDNA signal to different factors and allows for better interpretation of results from field sampling efforts. The goals of this work include: (1) modeling vertical profiles of eDNA concentrations and their temporal variability for migrating mesopelagic organisms; (2) understanding how biological and physical parameters affect the eDNA profiles; (3) demonstrating how a mechanistic model can be used to test ecological hypotheses; and (4) providing guidance for eDNA field sampling and interpretation.

## Methods

To capture the first-order characteristics of the distribution of eDNA shed by mesopelagic species, we used a mechanistic model with one spatial dimension (the vertical dimension). It treats migrating organisms as a continuous point source of eDNA and simulates temporal evolution of the eDNA concentration vertical profiles in a one-dimensional ocean with climatological conditions. Horizontal variation of the distribution of eDNA shed by mesopelagic species is not considered in this study. The point source in the model represents a number of individuals within a single species. The simulation includes relevant eDNA transport processes using a vertical Price-Weller-Pinkel (PWP) dynamical module^42^. We use this model to first explore the sensitivity of eDNA concentration profiles to a variety of biological and physical parameters and to assess vertical and temporal variability of the eDNA concentration. Table 1 outlines the parameters, separated by type, and ranges of values for each parameter used in the sensitivity analysis (see “Sensitivity analysis” section below) and in a case study of how the model can be used to determine the percent of individuals that migrate (see “Application to ecological questions” section below). We then discuss how the mechanistic model can be used in conjunction with field sampling to test ecological hypotheses, highlighting how analysis of eDNA concentration profiles can provide information regarding where and when organisms are located in the water column. Finally, we comment on implications of this work and how the model can be used to both design and interpret observations.

**Table 1:** Parameters separated by category.

Category	Parameter in model	Constant value used in model	Changes in sensitivity test?	Changes in percent migrate test?	Notes
Organism/eDNA	eDNA shedding rate	1 eDNA mass unit/time step	No	No	Still relatively unknown, likely varies with time (many scales from second to years) and by organism, maybe varies with temperature or other biotic/abiotic conditions
Organism/Ocean	Day depth	500 m	No	No	Varies with light/temperature, Varies with organism
Organism/Ocean	Night depth	50 m	No	No	Varies with light/temperature/food availability, varies with organism
Organism/Ocean	Migration times	Up: 2 h before sunset to 1 h after sunset; down: 1 h before sunrise to 2 h after sunrise; sunrise/sunset: determined by season	No	No	Varies with light/temperature/food availability, varies with organism
Organism	Thickness of “layer”	20 m	No	No	Varies with organism
Organism	Percent of individuals (within a species) that vertically migrate	N/A	Yes - 0, 50, 100%	Yes - 0, 10, 20, 30, 40, 50, 60, 70, 80, 90, 100%	Still relatively unknown
Organism/eDNA	eDNA size distribution	N/A	Yes - ratio of LP:SP - 1:1, 1:2, 2:1	No	Unknown, likely varies with time
eDNA	Settling rate	N/A	Yes - 0, 25, 50 m/day	No	Unknown, likely varies with eDNA form (use value based on literature for marine snow)
eDNA	Breakdown rate	0.19 1/h	No	No	Unknown (use value based on literature for fecal pellet)
eDNA/Ocean	Decay rate	N/A	Yes - 0.01, 0.1 1/h and k = 0.05 + 0.0014*T 1/h	No	Varies with temperature (possibly other biotic/abiotic factors and by organism) (Supplemental Fig. S3)
Ocean	Mixing profile	Determined by season	No	No	Varies by season and depth (Supplemental Fig. S1)
Ocean	Vertical advection profile	N/A	Yes - 0, 1E–4, −1E–4 m/s	No	Varies by season and depth (Supplemental Fig. S2)

### Model overview

#### Governing equations

To maintain simplicity and also provide a more realistic scenario where both settling and neutrally buoyant eDNA particles^28^ are considered, we assume that the eDNA material at any given time in the system consist of eDNA particles of two size classes. Here, large eDNA particles are on the order of 10 s of µm to 1 mm and represent materials such as fecal pellets, chunks of tissue, or gametes that would be subject to both settling and physical breakdown over time. Small eDNA particles are on the order of 0.1 µm to 10 s of µm and would be any eDNA shed from an organism that has a density such that they are near-neutrally buoyant including extracellular eDNA or forms such as sperm, urine, blood, or single cells. This size class of small particles is so small that we can neglect their settling. In practice, these two size fractions of eDNA would be captured using the common method of filtering water through a < 1 µm pore size filter^43^. Both small and large particles in the model are subject to the same eDNA decay rate constant and large particles break down over time into small particles.

The equation describing the change of eDNA concentration in the form of large particles is:

1 $$\begin{aligned} \frac{\partial {C_{LP}}}{\partial {t}} = - w_v\frac{\partial {C_{LP}}}{\partial {z}} + \frac{\partial {}}{\partial {z}}\left( \kappa _z\frac{\partial {C_{LP}}}{\partial {z}}\right) - kC_{LP} - \delta C_{LP} + w_s\frac{\partial {C_{LP}}}{\partial {z}} + \hat{S_{LP}} \end{aligned}$$

where $C_{LP}$ is the concentration of large eDNA particles, $w_v$ is the vertical velocity [L/T] with a positive value pointing upward, $\kappa _z$ is the vertical diffusivity [L$^{2}$/T], *k* is the first order decay rate constant [1/T], $\delta$ is the breakdown rate of particles [1/T], $w_s$ is the settling rate of eDNA [L/T], and $\hat{S_{LP}}$ is the shedding rate of new large eDNA particles.

Similarly, the equation describing the temporal evolution of the concentration of small particles of eDNA, $C_{SP}$, is:

2 $$\begin{aligned} \frac{\partial {C_{SP}}}{\partial {t}} = - w_v\frac{\partial {C_{SP}}}{\partial {z}} + \frac{\partial {}}{\partial {z}}\left( \kappa _z\frac{\partial {C_{SP}}}{\partial {z}}\right) - kC_{SP} + \delta C_{LP} + \hat{S_{SP}} \end{aligned}$$

where the parameters are the same as Eq. (1). Note that there is no settling term in Eq. (2) as small eDNA particles are assumed to be neutrally buoyant and that the $\delta$ term in Eq. (2) has the opposite sign as that in Eq. (1), reflecting eDNA volume conservation during the breaking-down of the large particles into small particles.

#### Physical model

The 1-D vertical model is implemented in MATLAB. The physical module is based on a previously published 1-D model of physical processes in the mixed layer^42^ and is modified to include the relevant organism and eDNA parameters. The model spans from the surface down to 1500 m deep with a vertical resolution of 0.5 m and has a time step of 10 s. Each simulation runs for a total of 90 days, representing one of the four seasons: summer (July, August, September - JAS), fall (October, November, December - OND), winter (January, February, March - JFM), and spring (April, May, June - AMJ). The model has a zero-gradient open boundary condition at the bottom. The model is forced on the surface by climatological, 8-hourly meteorological conditions from the NCEP/NCAR Reanalysis at a location in the Northeast Atlantic Ocean (37.047$^\circ$ N, -71.25$^\circ$ W). Inputs include air temperature, short wave radiation, long wave radiation, precipitation, air pressure, air humidity, and winds. The wind stress and heat fluxes are calculated using the bulk formulae^44^.

The initial vertical distribution of the temperature and salinity in the model are seasonal climatological profiles obtained from the NCEI World Ocean Atlas 2018 at a site in the Northwest Atlantic slope sea (39.125$^\circ$ N, −70.875$^\circ$ W; north of the Gulf Stream). The model also simulates the influences of vertical mixing and advection on the temperature, salinity and eDNA particles. The mixing influence is incorporated by prescribing synthetic vertical profiles of vertical diffusivity that combines observed seasonal mean mixed layer depth^45^ and simulated seasonal mean vertical diffusivity profile from an operational model^46^, both at a site in the Northwest Atlantic slope sea (see Supplemental Text S1 and Supplemental Fig. S1). Vertical advection profiles are set to increase linearly from 0 ms$^{-1}$ at the surface to the maximum value of ($10^{-4}$ ms$^{-1}$) at 200 m and then decrease linearly back to 0 ms$^{-1}$ at 400 m (Supplemental Fig. S2). This prescribed advection profile does not change seasonally and represents enhanced vertical motions associated with sub-mesoscale processes in the upper water column (e.g.,^47^). Note that the result of this study is not sensitive to the prescribed vertical profiles of diffusivity or vertical velocity (see below).

The physical parameters that will be adjusted in this study include: the initial temperature and salinity profiles, the mixed layer depth, the vertical diffusivity profile, and the vertical velocity profile (see Ocean parameters in Table 1).

#### Organism movement

**Figure 2:**
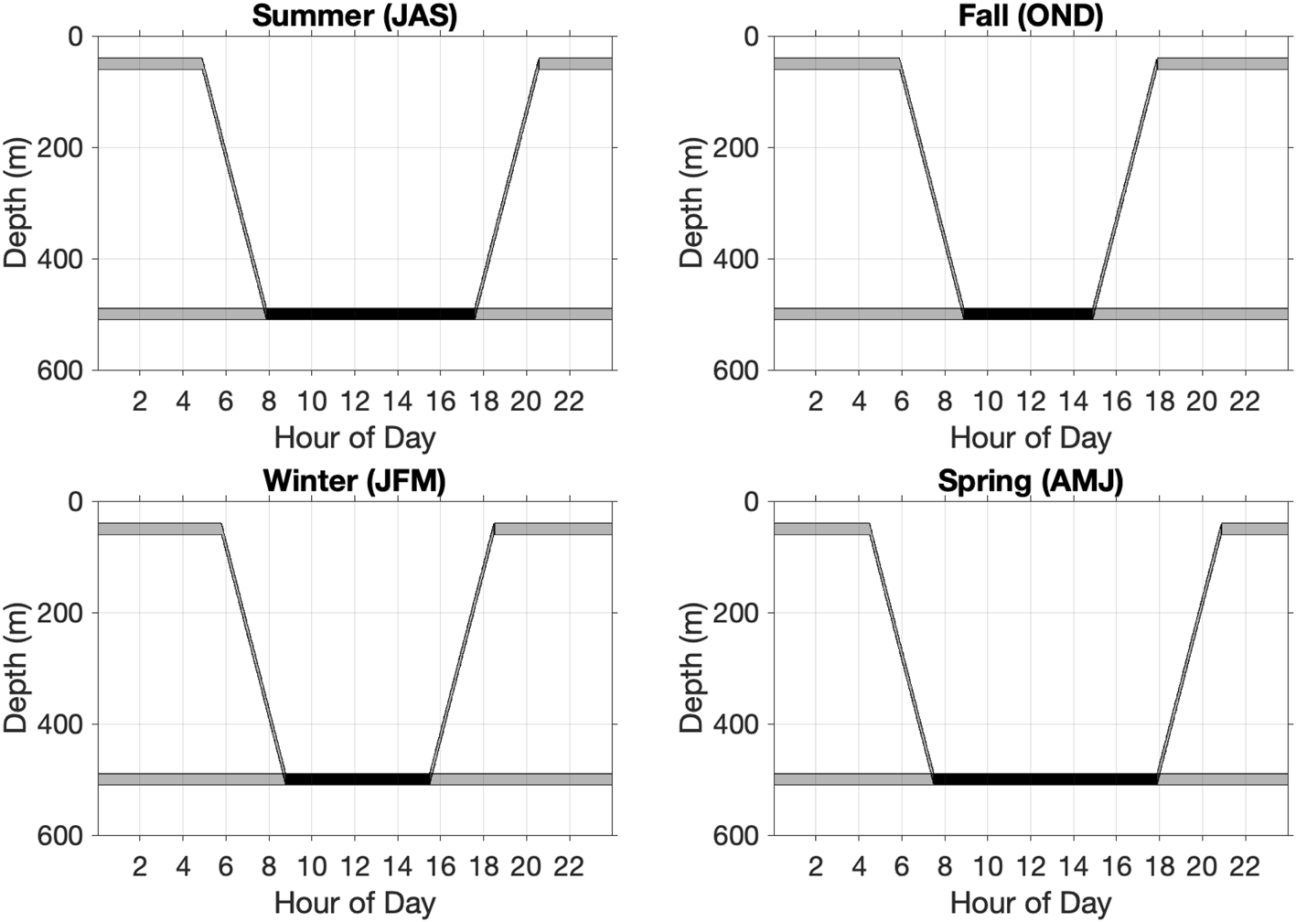
Representative organism migration curves for each season. Migration times change with sunrise and sunset for each season. This illustration assumes that 50% of individuals migrate. The shading shows the percentage of individuals with black indicating 100%, i.e., all organisms, and grey indicating 50%. These numbers vary among the simulations.

This study focuses on eDNA shed by vertically migrating organisms living in the mesopelagic ocean. For simplicity, we assume the organisms reside at the constant depth of 500 m during the day and the constant depth of 50 m during the night. These depths fall within the range of where migrating organisms are found at these times^41,48^. As the objective of this study is to develop a qualitative understanding of the vertical distribution of the eDNA concentration, the exact depth a particular mesopelagic species resides at in the daytime is not crucial. Organisms begin migrating to the surface two hours before sunset, and the upward migration ends one hour after sunset. The time of sunset and sunrise are determined seasonally using NOAA’s ESL solar calculator for the year 2019 at 42.35$^\circ$ N, −71.05$^\circ$ W. Both upward and downward migrations are assumed to be in constant speed, and the migration times do not change within each season (Fig. 2).

The migration parameters that can be adjusted in the model include: daytime residing depth, nighttime residing depth, the start and end times of migrations (and thus duration), the percent of individuals that migrate, and the layer thickness (i.e., “width” of school). (See Organism parameters in Table 1). In this study, we focus on the start and end times of migrations (and thus duration) and the percent of individuals that migrate.

#### eDNA parameters

We assume organisms are continuously shedding eDNA, and the shedding rate is fixed in time in each simulation. The eDNA then remains in the water column, where it is subject to transport and decay as described by Eqs. (1) and (2). Although several studies have characterized eDNA shedding rates of different marine organisms^29,49,50^, shedding rates are highly variable across studies and have high error associated with them, likely due to high temporal variability^29^. Here, we use a constant shedding rate of 1 mass unit per time step (10 seconds). Note that this study focuses on the *relative* vertical distribution of the eDNA concentration and its temporal variability, rather than its absolute value. The particle size distribution of eDNA (and the breakdown rate of large to small particles) and the settling rate of eDNA are largely unknown and expected to be time-varying^28^. Here we use a breakdown rate of fecal pellets to apply to large eDNA particles breaking down into small eDNA particles^51^. The most well characterized parameter of eDNA is decay rate, which several studies have characterized as a function of water temperature^29,49^. The upper and lower limits of the decay rate have also been well established^29,31^. Finally, there are currently no estimates of eDNA settling rates. We use values of marine snow settling rates^52^ to apply to large eDNA particles that are subject to settling in the model.

The eDNA-related parameters that are adjusted in this mechanistic model include: settling rate of large particles; ratio of large particles to small particles that are shed by an organism; eDNA breakdown rate (large particles to small particles); the eDNA decay rate. (See eDNA parameters in Table 1). Note that shedding rates in the model do not change over the course of each simulation.

### Sensitivity analysis

A series of 90-day simulations were carried out with altered values of the aforementioned parameters to examine the sensitivity of the eDNA vertical distribution to the parameters. Table 1 provides a list of the parameters that were adjusted in the sensitivity analysis. Note that the diffusivity profile (both the shape of the profile and the mixed layer depth), the temperature profile (which impacts the eDNA decay rate), and the daytime length (which impacted migration times) all change with the season. Other sensitivity parameters include vertical advection, settling rate of large particles, the ratio of large to small eDNA particles shed by organisms, and the percent of individuals that migrate. A total of 972 simulations were conducted with the following sets of parameters: 4 mixing profiles (one for each season), 3 vertical advection profiles (upwelling, downwelling, none), 3 settling rates of large eDNA particles, 3 decay rate constant scenarios (two constant values representing high and low values in the literature^31^, one temperature-dependent rate^29^ for each season, Supplemental Fig. S3), 3 scenarios for the percent of individuals that migrate, and 3 ratios of large to small eDNA particles shed by the organisms.

In order to assess the impact of the parameters on the eDNA profiles, several metrics were defined. First, the water column was divided into three sections: surface layer (0–100 m), mid-depth (100–450 m), and deep water (450–550 m), based on our representative daytime and nighttime depths (500 m and 50 m) used for the simulations. For each simulation, the mean and maximum eDNA concentration in each depth bin was recorded. Also, the cumulative eDNA concentration in each depth bin was normalized to calculate the proportion of eDNA in each depth bin at any given time during the simulation. For each simulation, the mean and standard deviation of the proportion of eDNA in each depth bin were calculated over the course of the 90 day simulation.

A first-order comparison of the vertical length scales of eDNA transport by different processes were conducted. Here, we use a time scale of eDNA decay, T$_{90}$, i.e., the time it takes for 90% of the released eDNA to decay, to determine the vertical length scale of each process. In particular, we would like to estimate, the vertical distances eDNA is transported by advection, mixing, and settling before 90% of the released eDNA has decayed, L$_{mix}$, L$_{advect}$ and L$_{settling}$, respectively. These quantities are defined mathematically as,

3 $$\begin{aligned} T_{90} = \frac{-ln(0.1)}{k_{avg}} \end{aligned}$$

4 $$\begin{aligned} L_{mix} = \sqrt{\kappa _v T_{90}} \end{aligned}$$

5 $$\begin{aligned} L_{advect} = w_{vm} T_{90} \end{aligned}$$

6 $$\begin{aligned} L_{settle} = w_s T_{90} \end{aligned}$$

where $k_{avg}$ is the average decay rate constant [T$^{-1}$] for the whole water column over the 90 day simulation, $\kappa _v$ is the maximum vertical diffusivity coefficient [L$^{2}$T$^{-1}$] , $w_{vm}$ is the maximum vertical velocity [LT$^{-1}$] , and $w_s$ is the settling rate [LT$^{-1}$]. Note that because *k* is temperature dependent, the value of the decay rate constant will change both vertically (e.g., deeper water will be colder and have a lower decay rate constant) and temporally (e.g., at a given depth, the water temperature will be warmer in the middle of the day and thus will have a higher decay rate constant).

### Application to ecological questions

After obtaining a first-order understanding of the eDNA concentration profile and the impact of the parameters on the eDNA distribution, we ran another set of model simulations to examine if vertical and temporal eDNA concentration variability can shed light on an ecological question regarding what percentage of individuals within the same species migrate on a daily basis. To do this, for each season, all parameters were held constant except for the percent of individuals that migrate, P$_{m}$. In each season, the value of P$_{m}$ varies from 0 to 100% with an increment of 10%. A total of 44 such simulations (4 seasons x 11 percent migrate scenarios) were carried out. We then compared mean and maximum eDNA concentrations in the surface layer (0–100 m) to those in the deep water (450 to 550 m), and examined the relationship between the surface-to-deep ratios and P$_{m}$.

## Results

### Overview and sensitivity analysis

#### eDNA concentration profiles

**Figure 3:**
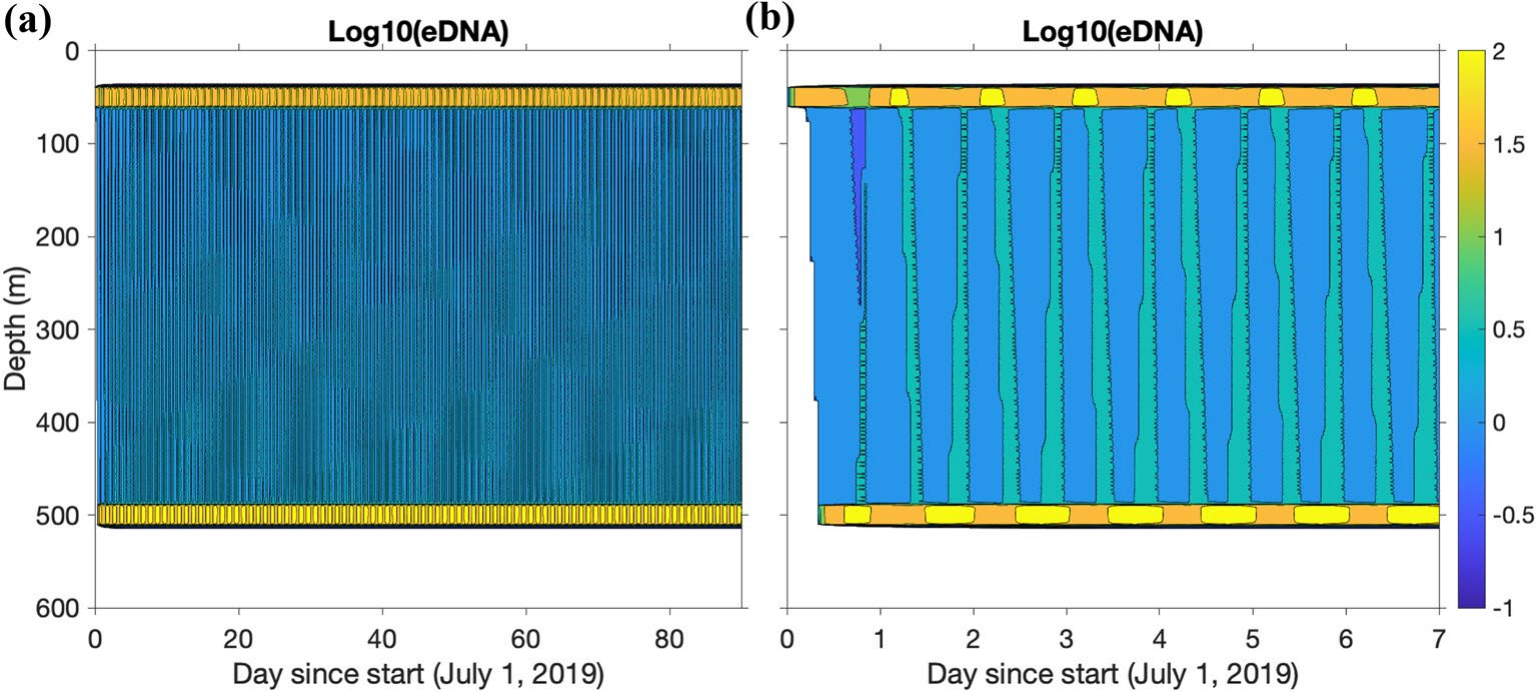
Temporal evolution of the eDNA concentration vertical profile in a representative simulation. Here, a summer (JAS) simulation with 50% of individuals migrating is shown. The eDNA concentration is log$_{10}$ transformed, and it is primarily a vertical bimodal distribution with peaks centered around the day depth (500 m) and the night depth (50 m). Diurnal variability (orange vs. yellow) induced by organism migration can be seen at 50 m and 500 m. Panel a) shows the whole 90-day simulation and panel b) shows the first 7 days of the same simulation.

In all simulations, the eDNA concentration profile stabilizes after just a few days. The resulting steady state eDNA concentration profile largely resembles a bimodal distribution with peaks centered on the daytime and nighttime residing depths (Fig. 3). Even though the organisms transit through all the intermediate depths during the migration, the time they spend at a particular intermediate depth (seconds to minutes) is much shorter than the time they spend at the surface or deep residing depths (several hours). This result suggests that, within the parameter space this study covers, eDNA cannot be transported a significant vertical distance by any of the physical processes, i.e., settling, mixing, advection (see below). This also indicates that the general vertical distribution of eDNA concentrations of a target mesopelagic species in the ocean can be quantified from just a single vertical profile of measurements taken at anytime of the day, and that both daytime and nighttime residing depths of the target species can be inferred from the profile.

**Table 2:** Depth bin metrics for Summer 50% migrate case.

Depth bin	Minimum (%)	Mean (%)	Maximum (%)	Standard Deviation (%)
Surface (0–100 m)	5.91	13.3	22.5	5.04
Mid-depth (100–450 m)	4.76	7.78	11.6	1.72
Deep (450–550 m)	69.4	78.9	88.3	5.63

Minimum, mean, maximum, and standard deviation of the percent of eDNA found in each depth bin over the course of a single simulation.

**Figure 4:**
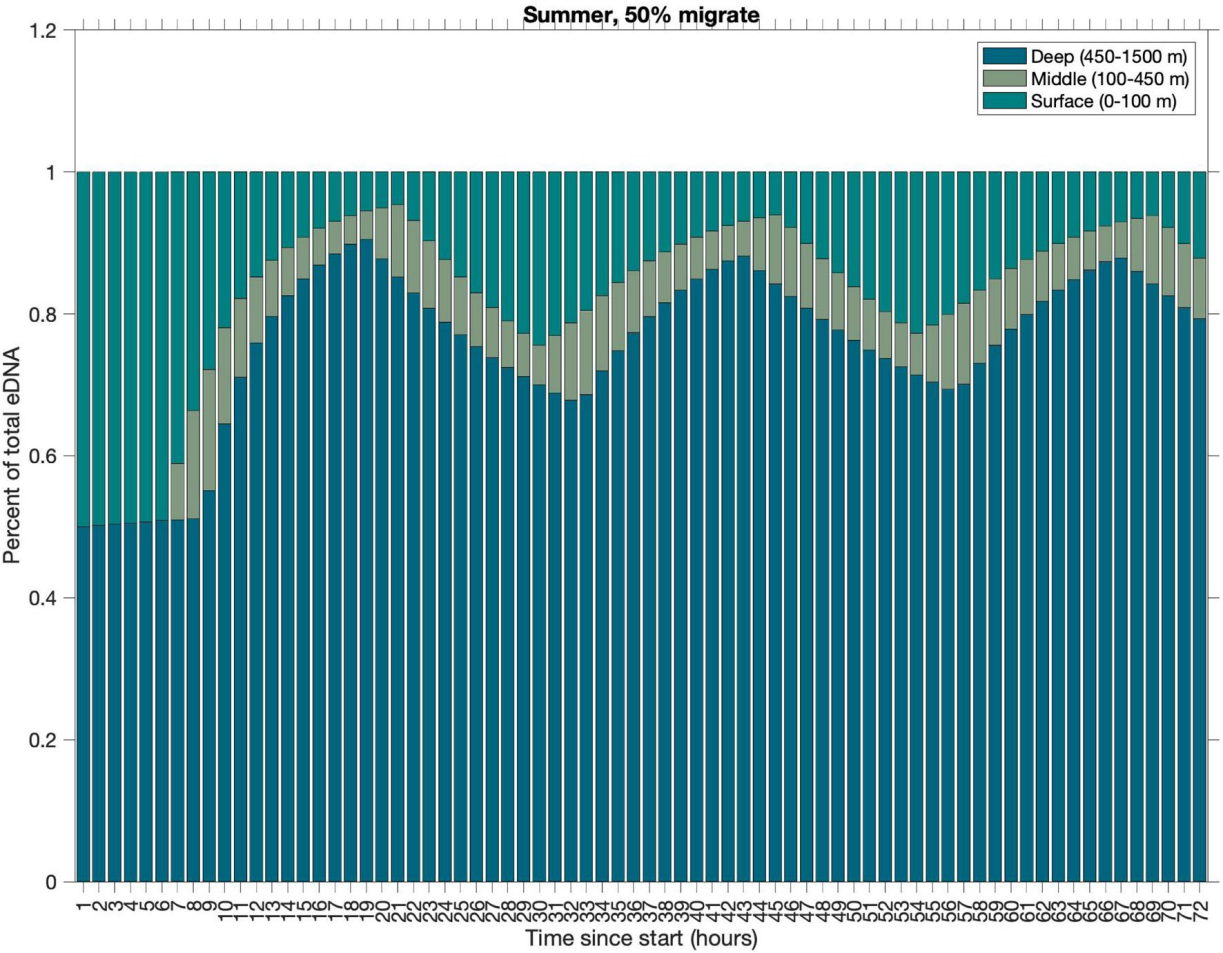
Temporal evolution of the proportion of eDNA concentration binned by depth category in a representative simulation. Here, the first three days (72 h) of a summer (JAS) simulation with 50% of individuals migrating is shown. The proportion of eDNA is shown in three depth bins at each time point: surface (0–100 m), mid-depth (100–450 m) and deep water (450–550 m). The DVM-induced temporal variation of the relative proportion and the persistence of eDNA in the surface layer when organisms are not present in the daytime are pronounced.

Because the eDNA concentration profiles largely resemble bimodal distributions, the water column can be simply divided into three layers (surface, mid-depth, and deep) to capture the different phases of the DVM and to characterize the eDNA vertical distribution. In this study, the layers are defined as 0–100 m, 100–450 m, and 450–550 m, respectively. The proportion of the simulated eDNA in the depth bins changes diurnally as organisms migrate up and down, but after a few initial migration cycles, the majority of eDNA stays within a single depth bin. For example, using the summer season with P$_{m}$ = 50%, the proportion of eDNA in each depth bin changes diurnally, but the largest proportion of eDNA remains in the deep layer, followed by the surface layer and then the mid-depth layer (Fig. 4, Table 2).

**Figure 5:**
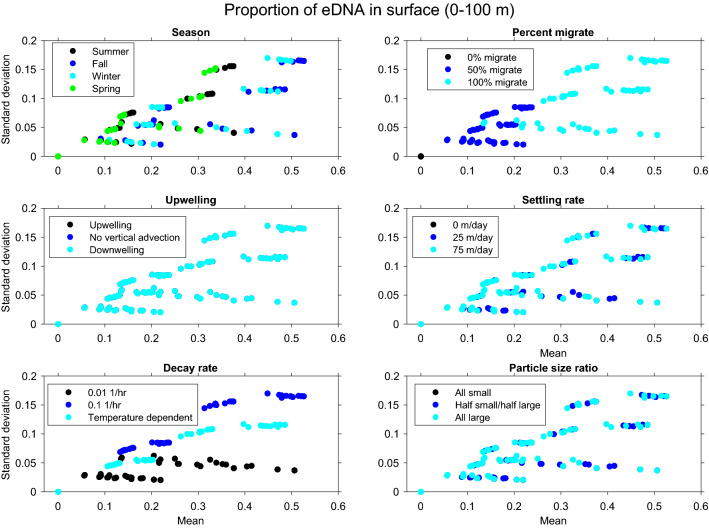
Standard deviation vs. mean proportion of eDNA found in the surface depth bin (0-100 m) for the sensitivity analysis of 972 simulations. All panels show the same data but colored by different parameters. Panels show the influences of seasons (summer, fall, winter, spring), percent of individuals that migrate (0, 50, 100%), vertical advection ($10^{-4}$ ms$^{-1}$, 0 ms$^{-1}$, $-10^{-4}$ ms$^{-1}$), settling rate (0 mday$^{-1}$, 25 mday$^{-1}$, 75 mday$^{-1}$), eDNA decay rate (0.01 h$^{-1}$, a temperature dependent relationship, or 0.1 h$^{-1}$,), and the ratio of small to large particles (all small, half small/half large, all large).

The eDNA vertical distribution changes with biological and physical parameters. For example, we find that as the season changes (and thus the mixing profile, temperature profile, and migration timing change), eDNA concentration in the surface layer changes (Supplemental Fig. S4). However, the changes induced by physical parameters (i.e., mixing, advection) are much less than those induced by the biological and eDNA parameters (e.g., percent of individuals migrating). This is demonstrated by the higher mean and standard deviation of the proportion of eDNA found in the surface layer when the 100% of individuals migrate rather than 50% or 0% (Fig. 5 and Supplemental Figs. S5 and S6).

#### Physics-induced vertical redistribution of eDNA

This section aims to quantify the vertical redistribution of eDNA induced by physical processes. For that, we apply representative values of the parameters in Eqs. (3)–(6) to obtain the vertical distance that advection, mixing, and settling can redistribute eDNA in the time that it takes for 90% of released eDNA to decay. Applying a temporally and spatially averaged decay rate constant of 0.06 h$^{-1}$ for the summer season, Eq. (3) gives $T_{90}$ = 38 h. Applying typical values of $\kappa _{v}$ = 10$^{-3}$ m$^{2}$s$^{-1}$, $w_{vm}$ = 10$^{-4}$ m s$^{-1}$, $w_s$ = 10 m day$^{-1}$ in Eqs. (3)–(6) gives the length scales for mixing, advection, and settling, $L_{mix}$ = 12 m, $L_{advect}$ = 13 m, and $L_{settle}$ = 16 m, respectively. Note that settling could only displace eDNA downward, while mixing and advection could displace eDNA either upward or downward.

The estimated settling distance has a relatively large uncertainty as there are no published estimates of eDNA settling rates or breakdown rates in the literature. Here we use information from fecal pellets and marine snow^51–54^, but it is unclear how applicable the values are to eDNA. For instance, some estimates of the settling rates of fecal pellets are faster than the value used in the model^51^, which means the settling distance of eDNA we provided is an underestimate. There is also little information about the particle size distributions of eDNA in the literature. All previous studies indicate that particle size distributions can vary significantly from time to time, likely related to the intermittent shedding of different forms of eDNA particles (e.g., cells, gametes, fecal pellets) by the organisms^29,50^. To really understand the impact of settling on eDNA distribution, it is necessary to know the proportion of eDNA being shed by an organism at a given time in the form of fecal pellets, subject to fast settling, versus extracellular material that is neutrally buoyant. However, it should be noted that the estimated settling length scale ($L_{settle}$) of 16 m does not account for the large particles breaking down over time in the model, which tends to reduce their settling rate. When the breakdown of large eDNA particles to small eDNA particles is considered, the estimated settling length scale will be even smaller (See Supplemental Text S2).

### Percentage of migrating individuals

The proportion of eDNA in each depth bin changes with both the percent of individuals migrating (P$_{m}$) and season. For a given season and a particular value of P$_{m}$, the temporal change of the percent of eDNA found in each vertical layer is relatively small with the standard deviation in the surface layer of 5.55%, mid-depth layer 1.88%, and deep layer 5.49%. As P$_{m}$ increases from 0 to 100%, the proportion of eDNA found in the surface layer increases, and that in the deep layer decreases, reflecting the increasing number of individuals moving up to the shallow water each night. When 0% of organisms migrate, no eDNA is found in the surface or mid-depth layers as the organisms remain at the nighttime depth at all times. In the summer simulations, when 100% of organisms migrate, the time-averaged proportion of eDNA in the surface layer is 33%, compared to 49%, 47%, and 31% in fall, winter, and spring, respectively (Table 3). Consistently, the percentage of eDNA in the deep layer also varies with the season. This seasonal influence on the eDNA depth distribution is primarily due to the different day lengths in each season. In fall and winter, the night is longer and thus organisms remain at the surface for longer. The water temperatures in the surface water are also colder in fall and winter, and thus eDNA persistence times are longer. However, more detailed examination of the model result shows that the change in time spent at depth versus surface due to the daytime length change is the primary driver of the seasonal differences in eDNA distribution (Supplemental Fig. S7).

**Table 3:** Simulated percent of eDNA found in surface layer (0–100 m) for different percent of individuals migrating in all seasons.

Season | % Migrate	0 (%)	10 (%)	20 (%)	30 (%)	40 (%)	50 (%)	60 (%)	70 (%)	80 (%)	90 (%)	100 (%)
Summer (JAS)	0	2.3	4.8	7.4	10	13	17	20	24	28	33
Fall (OND)	0	3.7	7.5	12	16	21	25	31	36	42	49
Winter (JFM)	0	3.5	7.4	11	16	20	25	30	35	41	47
Spring (AMJ)	0	2.1	4.4	6.8	9.5	12	15	19	22	26	31

Summer includes July, August, and September (JAS); fall includes October, November, and December (OND); winter includes January, February, and March (JFM); and spring includes April, May, and June (AMJ).

**Figure 6:**
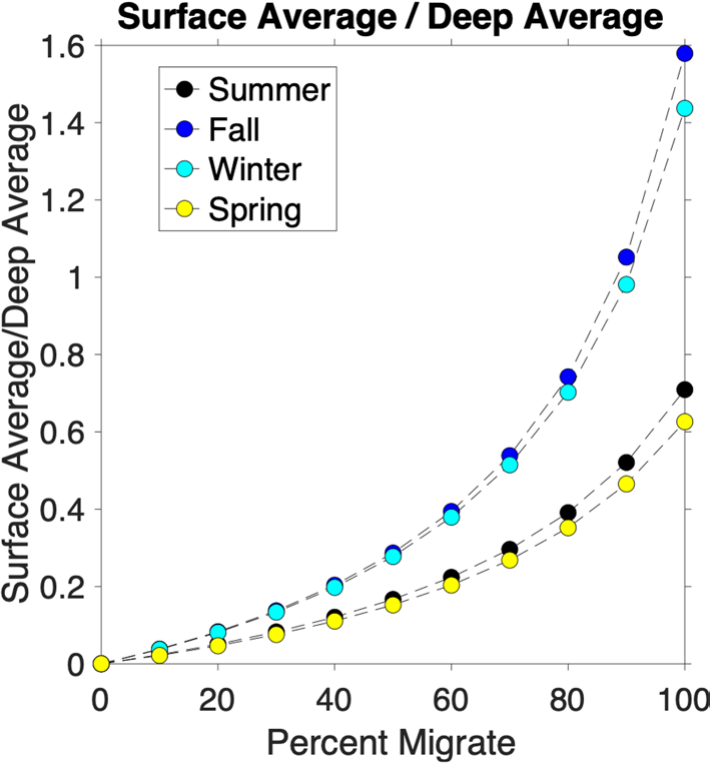
Ratio of average eDNA concentration in the surface to deep layers as a function of percent of individuals that migrate. The x-axis shows the percent of individuals that migrate. The y-axis shows the ratio of the average eDNA concentration in the surface layer (0-100 m) to the average eDNA concentration in the deep layer (450-550 m) for each season.

In all seasons, the model simulations show a one-to-one positive relationship between the ratio of modeled average eDNA concentration in the surface layer to that in the deep layer, C$_{s}$/C$_{d}$, and the percent of individuals migrating, P$_{m}$ (Fig. 6). This relationship is potentially useful for quantifying the percent of migrating individuals in the real ocean.

## Discussion

### General findings

A major finding of this mechanistic model is that the depth at which eDNA is detected corresponds closely to the depth at which the organism shed the eDNA. In the parameter space that we have explored, physical processes, such as mixing, advection, and settling, can displace eDNA vertically by only 10–20 m from the depth at which it was shed. This insight is critical for interpreting results from eDNA field measurements, especially to close the gap of inferring where organisms were located with respect to where eDNA was detected.

We demonstrate that we can use the variability in eDNA concentrations at certain depths to answer ecological questions regarding the vertical migration of species. For instance, measured changes of eDNA concentration at a certain depth can also be used to infer the times at which a species arrives at or leaves that depth. It is important to note that in the model we are measuring relative changes of eDNA concentrations, not absolute eDNA concentrations. Therefore, when applying the results to field samples, the model is most similar to results as measured by quantitative PCR (or similar methodologies) where eDNA from a single species is being measured in a quantitative manner. Results from metabarcoding in some cases suggest that the proportion of sequencing reads scale with species abundance, but there are several factors that need consideration when interpreting metabarcoding data, including taxon-specific primer affinity during PCR amplification, depth of sequencing, and other laboratory and bioinformatic challenges^55–58^. More simulations should be conducted with refined estimates of parameter spaces as information becomes available, particularly for the largely unknown parameters such as eDNA shedding and settling rates.

**Figure 7:**
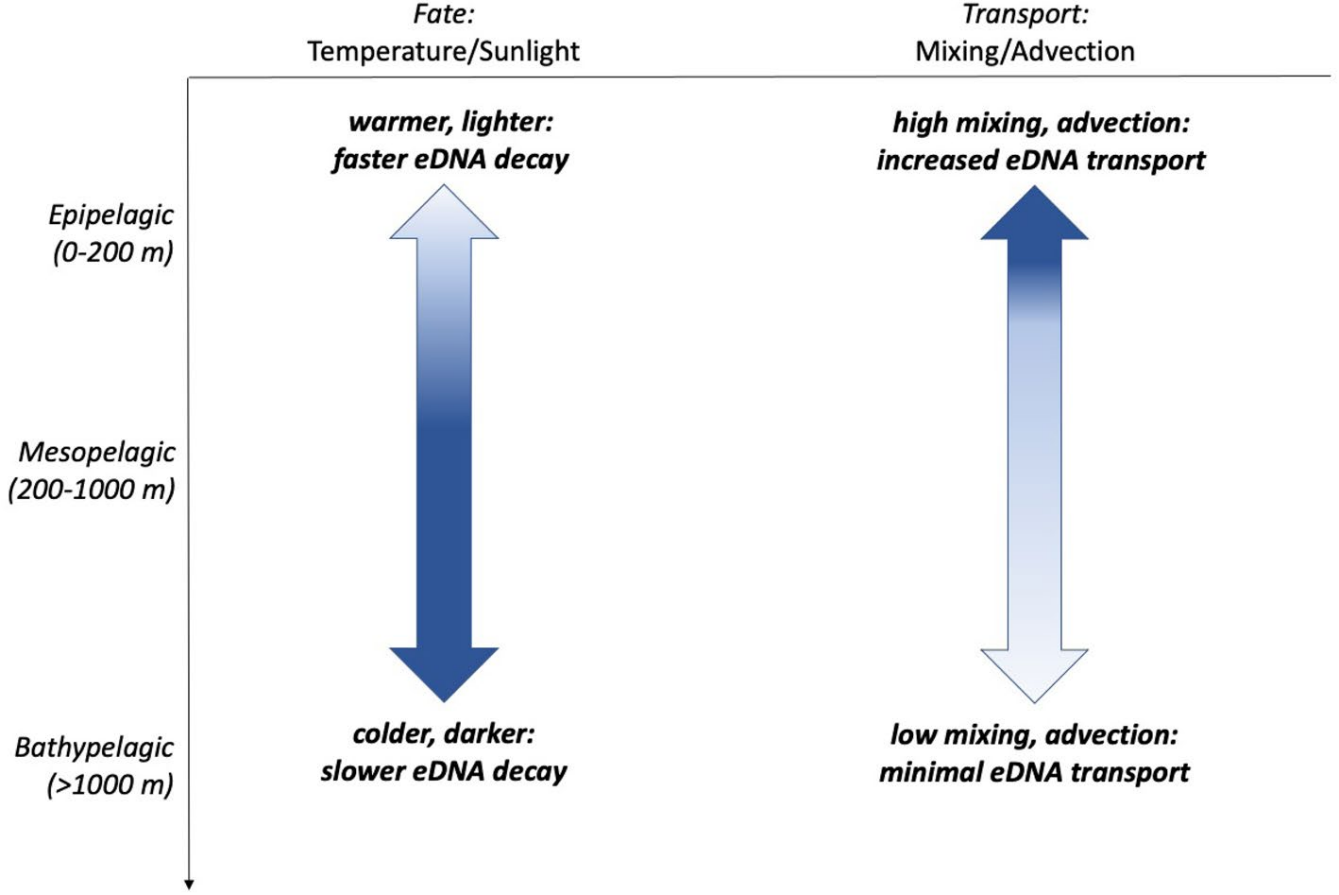
Variation of the influence of different processes at different depths of the water column. Y-axis shows depths from the mechanistic model. The left arrow shading indicates higher temperatures and more sunlight in surface waters transitioning to lower temperatures and no sunlight in deeper water. The right arrow shading indicates higher velocities and mixing in the water column in surface waters transitioning to low mixing and advection in deeper water.

We also emphasize that it is important for researchers collecting water samples for eDNA analyses to consider the biological and physical processes relevant at different depths in the water column (Fig. 7). For example, in shallower water (in the surface mixed layer), mixing will be more important than in deep water^44^, resulting in potentially larger contribution of vertical transport in regulating eDNA vertical distribution. Similarly, in regions of warm surface waters, eDNA persistence times will vary vertically, causing shorter persistence (and thus reduced transport) in the warm surface layer than in the cold deep water. Considering the impact of these parameters on the eDNA distribution over the whole water column over the time scale of days to season, our mechanistic model suggests that many of these processes have minimal impacts on the eDNA concentration profiles; but this modeling result should be tested with field data. Nevertheless, when designing an eDNA sampling scheme and interpreting eDNA data, it is important to consider the spatial and temporal scale of sampling, the range of relevant eDNA and physical parameters, and the influences from organism behaviors, the physical environment, and the decoupling of eDNA from the organism itself.

### Application to the mesopelagic ocean

The findings of this study are especially useful for under-explored habitats such as the mesopelagic ocean. There are numerous uncertainties in the underlying ecology in the mesopelagic, and multiple methods need to be used to characterize and understand the complex, difficult to access, and extremely large ecosystem. Using eDNA methods to gain insight on the mesopelagic is promising because of the ease of collecting water samples, the non-invasive nature, and the capability of detecting and differentiating a variety of species.

A small number of studies have analyzed water samples taken from the mesopelagic for eDNA signals^11,12,14,59^, citing detection of expected migrating and non-migrating taxa via eDNA metabarcoding. For example, Easson et al., 2020^59^ sampled water in the Gulf of Mexico for eDNA analysis in conjunction with acoustic sampling and found that vertical eDNA profiles (as measured in the percent of sequencing reads) for higher-level, primarily invertebrate, animal taxa did in fact change with movement of sound scattering layers as detected by acoustics. Also, importantly, taxa that are too small to be detected acoustically were identified by eDNA analyses. Similarly, Canals et al. 2021^60^ found high fidelity between expected mesopelagic fish migration patterns in the Bay of Biscay, France, and the relative proportion of eDNA sequencing reads, with increased eDNA read abundances of migratory fish species in the surface water samples collected at night and increased eDNA read abundances in deep water samples collected during the day. In the Northwest Atlantic, Govindarajan et al., 2021^11^ sampled water at various depths in the mesopelagic ocean (guided by acoustic data) over the course of multiple days and nights and found that different invertebrate taxa were detected by eDNA at different depths in the water column. However, the metazoan communities as detected by eDNA were more similar to one another based on the depth of sampling rather than the time of day when the water was collected. This suggests that even if organisms are migrating, there is a residual eDNA signal at the primary depth at which a species resides that does not completely disappear even if the organism has migrated away. Yet none of the above datasets suggest a major influence of physical transport on the eDNA distribution, which is consistent with with our results from the mechanistic model. Higher-resolution sampling and pairing results with a mechanistic model such as the one presented here would help to further interpret the eDNA signals detected in the previously mentioned studies.

The results here provide a first-order understanding of the vertical transport of eDNA signals representative of idealized scenarios in a particular parameter space, providing support for the observational studies citing distinct shifts in taxa as measured by eDNA found in moving acoustic scattering layers^11,59^. The modeling analysis of the vertical distribution of eDNA shed by a mesopelagic, diel-vertically migrating organism presented in this study shows that it takes only a few days for the eDNA concentration profile to reach a quasi-equilibrium state. The variability in eDNA vertical distribution induced by the changes in water temperature and the associated changes in decay rate constant over the course of a season (90 days) is relatively small. This implies that ecologically, after organisms move in a repeated DVM pattern over just a few days, the basic pattern of the eDNA concentration profile of the species will remain stable and thus can be used to infer the residing depths of the organisms during the day and night (assuming that the migration pattern and the depths at which the species resides at day and night remain largely consistent). Meanwhile, changes of the eDNA concentration in the surface and deep layers during the dawn and dusk can be used to estimate the times at which organisms begin and end daily migrations. The concentration of eDNA in the surface will continue to increase as organisms remain in the surface but will begin to decrease as soon as the downward migration begins. Due to the mixture of the signals from different species, it is often unclear from either acoustics or net tows which mesopelagic species start migrating at a given time but changes in eDNA concentrations could be used to answer this question. Furthermore, if organisms shift their day depth over the time scale of a few days, for example, the change would be detected in the changes to the eDNA concentration profile. More studies are needed to further improve our knowledge on the parameters and processes that could potentially affect the eDNA distribution in the real ocean.

### Practical applications

The modeling analysis presented here also demonstrates the potential for eDNA field sampling to be conducted in conjunction with other methods (i.e., nets, acoustics, and modeling) in order to accelerate the understanding of ocean habitats^11,12,59^. It is advantageous to pair observational data with the model on both field sampling design and field data interpretation and to integrate eDNA data with traditional sampling methods, as no one method can provide a complete picture of biodiversity on its own. Here, we outline a few ecological questions that can be addressed based on our findings with this mechanistic model.

- **Refining the depth range at which a target species resides at a given time of day.** Conduct high-resolution sampling above, within, and below a target depth and plot eDNA concentration profile as a function of depth. Based on the mechanistic model, the depth with the greatest eDNA concentration (as measured by quantitative PCR or similar) should be the depth at which the species primarily resides.
- **Providing taxonomic resolution for acoustic biomass data and determining specific migration times of specific species.** Acoustic data can determine when the biomass corresponding to a particular acoustic frequency migrates up and down each night and day. But the timing of the migrations may be species-specific. There are often early and late migrators in the group (i.e., some organisms start migrating up or down earlier than others)^41^. With acoustic methods it is not yet possible to determine which organisms are migrating early or late. By sampling water before, during, and after an upward or downward migration, the variability in eDNA concentration for a target species (or multiple species) can be used to determine migration times and sequence. Similarly, eDNA analysis based on water samples inside and outside of distinct layers could be used to determine the taxonomic composition of the layers.
- **Determining what percentage of individuals within a single target species migrate.** Here, we demonstrate a one-to-one positive relationship between the ratio of modeled average eDNA concentration in the surface layer to that in the deep layer, C$_{s}$/C$_{d}$, and the percent of individuals that migrate, P$_{m}$ (Fig. 6). C$_{s}$/C$_{d}$ can be calculated from high-resolution vertical profiles of measured eDNA concentrations. Therefore, these measurements together with the simulated relationship can provide estimates of P$_{m}$. Thus, the changes to the ratio (C$_{s}$/C$_{d}$) over time in observational data should reflect the movement (or lack of movement) of individuals within a species. For example, if C$_{s}$/C$_{d}$ increases just after sunset, some percent of individuals of the target species has migrated to the surface. Though this requires information on the physical and biological parameters in the model, which are difficult to accurately estimate, the relative ratios are still useful to determine the relative change in P$_{m}$ assuming the other parameters do not change dramatically, or changes of the parameters do not dramatically affect the modeled relationship between C$_{s}$/C$_{d}$ and P$_{m}$. For example, if 50% of organisms migrate during one period and 90% during another period, the measured C$_{s}$/C$_{d}$ in those periods should differ significantly. The measured change in C$_{s}$/C$_{d}$ can thus be combined with the modeled relationship between C$_{s}$/C$_{d}$ and P$_{m}$ to retrieve information on the changed species migration behavior, even though the absolute value of the percent individuals migrate remains unknown. Similarly, the model relationship and field measurements could be combined to investigate changes of the species migration behavior in different geographic locations or induced by other biotic and abiotic factors (e.g., thermal preferences of organisms). These inferences from eDNA concentrations can provide more information than is currently available and help to direct future efforts with other tools like nets and acoustics.

In the future, if technologies for sampling water for eDNA analyses advance beyond current capabilities, eDNA can be used to address a greater variety of ecological questions. Especially for regions of relatively low biomass, it will be advantageous to be able to sample larger volumes of water for eDNA analyses over broader spatial scales and even collecting water samples over integrated depths (i.e., more similar to a net tow) rather than at discrete depths. Also, previous studies show substantial variability between biological replicates of water samples from 5-10 L Niskin bottles, suggesting that these sampling volumes aren’t adequately matched to the scale of eDNA distribution^61^. Autonomous vehicles and other platforms (e.g., Mesobot^4^ and 3G-ESPs^62^) should be explored for adaptive water sampling, in particular for higher resolution spatial and temporal sampling schemes and increased water volumes, in order to take full advantage of the insights from the modeling.

### Limitations and areas of future research

We emphasize that there remains uncertainty in many eDNA parameters, especially as many are likely species-specific^29^. The parameters used in this study can be adjusted as more information becomes available. This study focuses on a small portion of the vast parameter space; however, our parameter choices are grounded in previously reported values and are realistic for our study system. While we did not quantify how greater variability in the parameter values would affect the modeling outcomes here, we demonstrate here that even with the uncertainties, our results show that eDNA analysis is a useful tool for understanding the mesopelagic ecosystem. Moreover, additional parameters could be included in the model. For example, predation and movement of prey eDNA via predators is not currently included as a source (or sink) of eDNA^63^. Redistribution of prey eDNA via predator fecal matter could affect the vertical distribution of eDNA in the water column if the predator migratory behavior is different than that of the prey. Future iterations of modeling efforts could include this additional parameter and the sensitivity to the eDNA variabilities.

Areas for future eDNA research that would be particularly useful are more information on shedding rates and settling rates. In particular, studies have shown that eDNA shedding rates can have high intra-individual, intra-species, and inter-species variability, and can be temporally variable across a range of scales^29^. For example, a given organism might shed at a different rate minute to minute based on a biological activity such as defecating or swimming^64^, or year to year based on life stage^65^. Future work should focus on bounding these variabilities and performing more simulations to determine the impact of varying shedding rates on eDNA concentration profiles.

In terms of settling rates, even though we expect settling rate is likely unimportant for the majority of eDNA sources, settling rates of particular forms, such as fecal pellets, could be high^54^, and this could result in large vertical transport distances. As demonstrated here, mechanistic models of eDNA distribution could help direct future eDNA research and prioritize processes that have greater impacts on our understanding of the distribution, transport and fate of eDNA particles in the ocean.

This mechanistic model provides a first-order but important framework for interpreting how eDNA signals relate to biomass and species distributions in the ocean. Here, we account for organism and eDNA movement within a 1-dimensional physical model of the mesopelagic ocean and determine how eDNA concentration profiles vary in the vertical direction and in time. The fate and transport of eDNA in the 3-dimensional ocean can be complex, especially in the mesopelagic ocean where organisms are migrating hundreds of meters every day and ocean currents and environmental conditions vary both horizontally and vertically. Careful consideration of these variabilities in space and time can shed light on how to relate eDNA concentrations to organism location and abundance. Due to the limited amount of field measurements available, a more comprehensive model simulating the variabilities is essential to making further progress on interpreting measured eDNA signals in the ocean, particularly to answer ecological questions about an understudied habitat such as the mesopelagic ocean.

## Supplementary Information

Supplementary Information.
