# Supplementary material for: Modeling characterization of the vertical and temporal variability of environmental DNA in the mesopelagic ocean
Source: Sci Rep. 2021 Oct 28;11:21273. doi: 10.1038/s41598-021-00288-5 (PMC8553870; doi:10.1038/s41598-021-00288-5)
Supplement: Supplementary file 1 — Supplementary Information. [file 41598_2021_288_MOESM1_ESM.docx]

**Supplemental Text S1.**

**Vertical diffusivity profiles**

The eDNA tracer, temperature, and salinity in the ocean are subject the influence of vertical mixing. To consider this in the one-dimensional model, we prescribe a profile of vertical diffusivity in each of the seasonal simulations. To obtain a representative mixing profile for each season, we combine data from i) a seasonal climatology of surface mixed layer depth and ii) a Regional Ocean Modeling System (ROMS)-based operational ocean model of the Northwest Atlantic slope sea (Wilkin et al., 2018). The mixed layer depth climatology was computed based on historical temperature and salinity vertical profiles measured by ARGO floats in the slope sea (Holte, et al., 2017). Values of the mixed layer depth in each season at (39.125ºN, 70.875ºW) are first extracted from the climatology. The idealized vertical diffusivity profile in each season is then designed based on the mixed layer depth information and the vertical pattern of the seasonal diffusivity climatology at (39ºN, 70ºW) given by the ROMS simulation in the period of 2007-2018. In particular, following the result of the operational model, the vertical diffusivity coefficient is set at 10^-3^ m^2^ s^-1^ in the surface mixed layer, and it decreases gradually to 10^-5^ m^2^ s^-1^ at depth following a vertical hyperbolic tangent function. The idealized vertical diffusivity profiles are shown in Fig. S1. Consistent with the ARGO measurements, they show the shallowest mixed layer depth in summer followed by spring, fall, and then winter.

**Supplemental Text S2.**

**Settling Rate and Breakdown Rate**

We consider the settling and breakdown of eDNA particles to assess their combined influence on the eDNA vertical distribution. Modelling an eDNA particle as a sphere with mass $m$ and diameter $d$, it will settle according to its Stokes settling velocity $w_{s}$ given by the expression (Stokes, 1851):

$$w_{s}=\frac{\left( \rho_{p}-\rho\right)}{18\mu}gd^{2},$$

where $\rho_{p}$ is the density of the particle, $\rho$ is the density of the seawater, $\mu$ is the dynamic viscosity of seawater and $g$ is gravitational acceleration. The mass of the particle is $m=\frac{\rho_{p}\pi d^{3}}{6}$. Assuming the particles break down (lose mass) with a first-order decay rate coefficient $\delta$, we can write an expression for $m$ over time $t$, given the particle’s initial mass $m_{0}$:

$$m=m_{0}e^{-\delta t}.$$

Using these relations, we can express the temporal evolution of the particle settling rate, given initial settling rate $w_{s}^{0}$ at time $t=0$:

$$w_{s}(t)=w_{s}^{0}\exp\left( -\frac{2}{3}\delta t \right)$$

Finally, we assume that the particle is moving vertically according to its settling rate such that $\frac{dz}{dt}=w_{s}$, where $z$ is the vertical position of the particle. This allows us to integrate our expression for $w_{s}\left( t \right)$ from $t=0$ to $t=\infty$ to find the maximum vertical distance that the particle will settle: $L_{\infty}.$

$$L_{\infty}=\int_{0}^{\infty} w_{s} dt=\frac{3}{2}\frac{w_{s}^{0}}{\delta} .$$

This indicates that, when accounting for changes in $w_{s}$ as the particle breaks down, the maximum distance the particles will settle, $L_{\infty}$, is 1.5 times the initial settling rate divided by the breakdown rate. In the model presented in this work, only the large eDNA particles are allowed to settle. Applying a representative settling rate of $w_{s}^{0}=10$ m day^-1^ and a representative break down rate of $\delta=.19$hr^-1^ in the formulae above, we obtain a maximum settling distance of $L_{\infty}=3.3$ m. This means that the particles will only settle 3.3 m vertically before they break down. This distance is an order of magnitude smaller than the predicted settling length scale $L_{settle}$= 16 m in the main text that does not take particle breakdown into account.


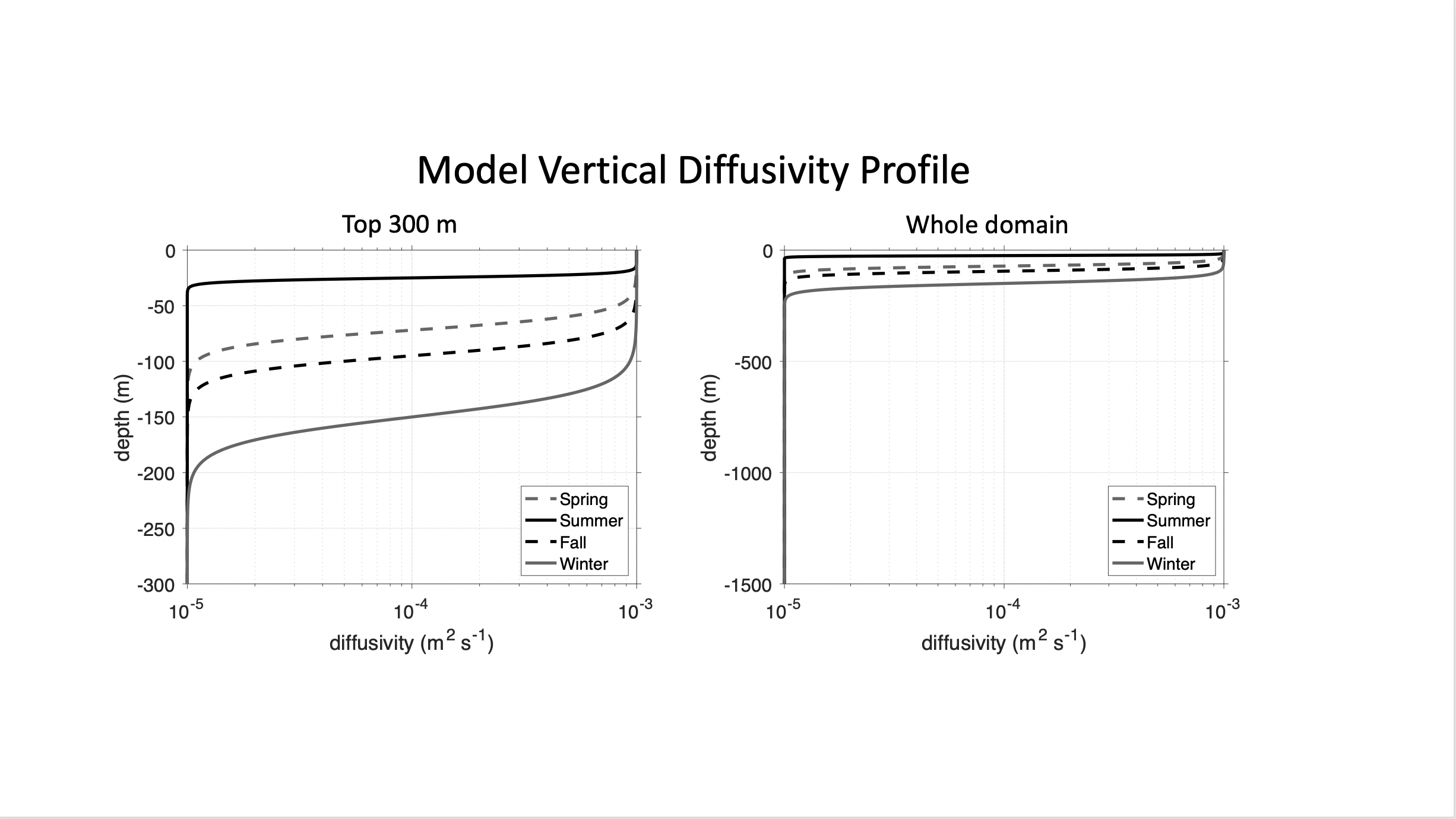


B)

A)

**Supplemental Figure S1. Model vertical diffusivity profile.** Prescribed vertical diffusivity profiles by season in A) top 300 m and B) the entire depth range of the model. Note that x-axis is on a logarithmic scale.


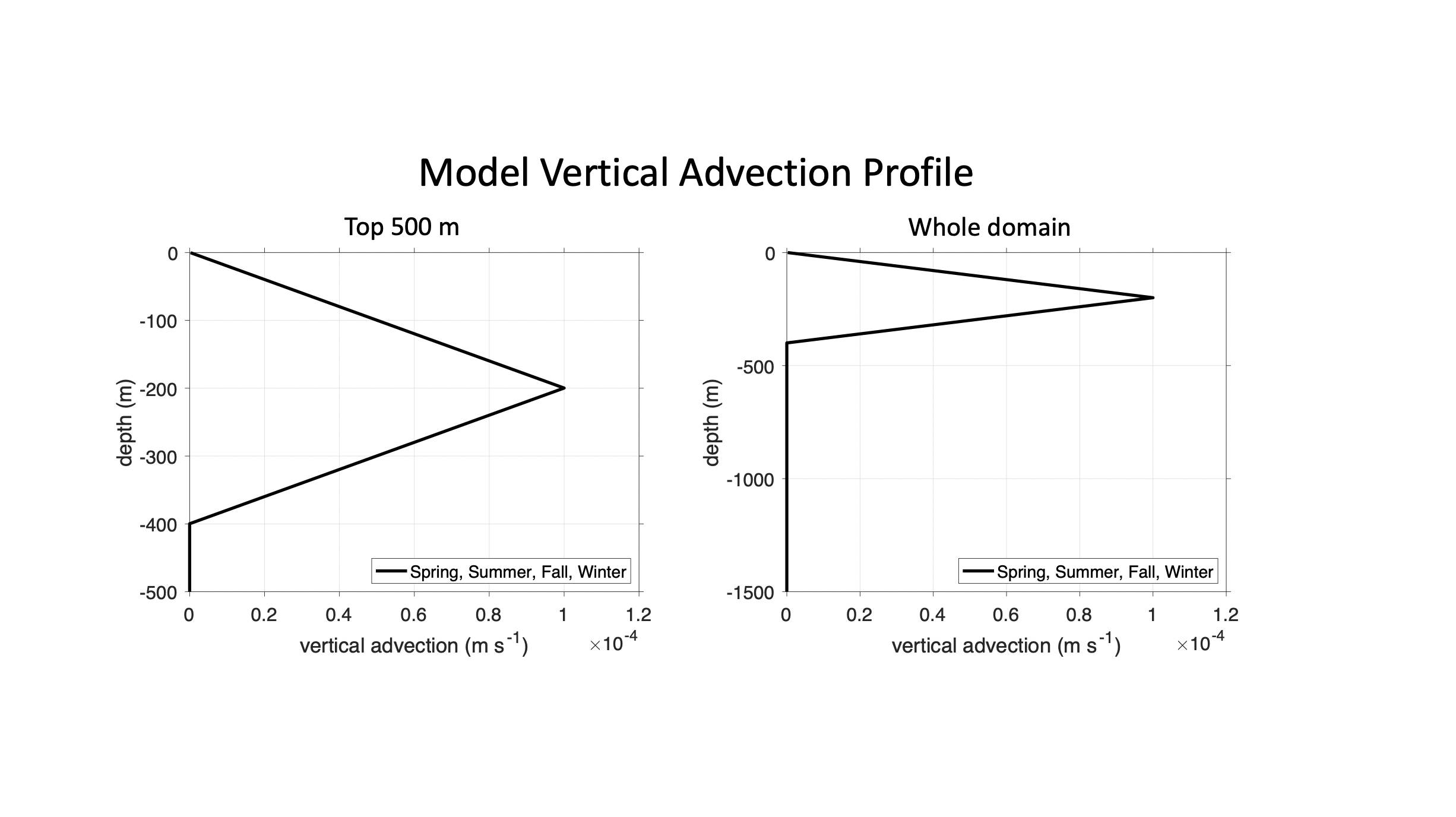


A)

B)

**Supplemental Figure S2. Model vertical advection profile.** Prescribed vertical profile of advection speed in A) top 500 m and B) the entire depth range of the model. The profile is the same for all seasons and linearly increases from 0 m s^-1^ at the surface to a maximum of 1 x 10^-4^ m s^-1^ at 200 m depth and then linearly decreases to 0 m s^-1^ at 400 m depth. In simulations, vertical advection can either be positive or negative depending on the upwelling or downwelling scenarios.


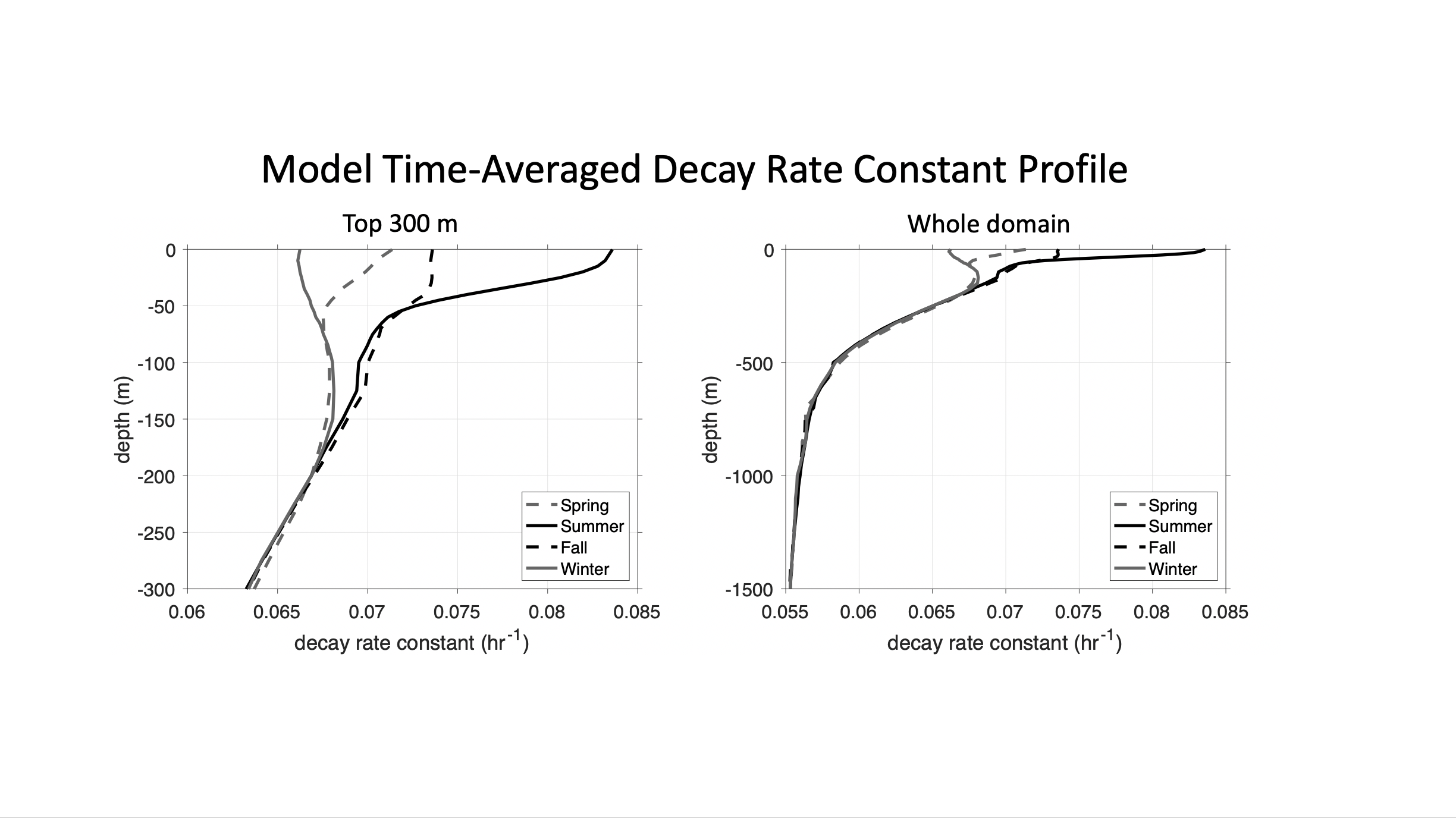


A)

B)

**Supplemental Figure S3. Model mean decay rate constant profiles.** Time-averaged decay rate constant profiles over the 90-day simulations by season in A) top 300 m and B) the entire depth range of the model. Decay rate constant is a function of water temperature and thus varies with depth and time.


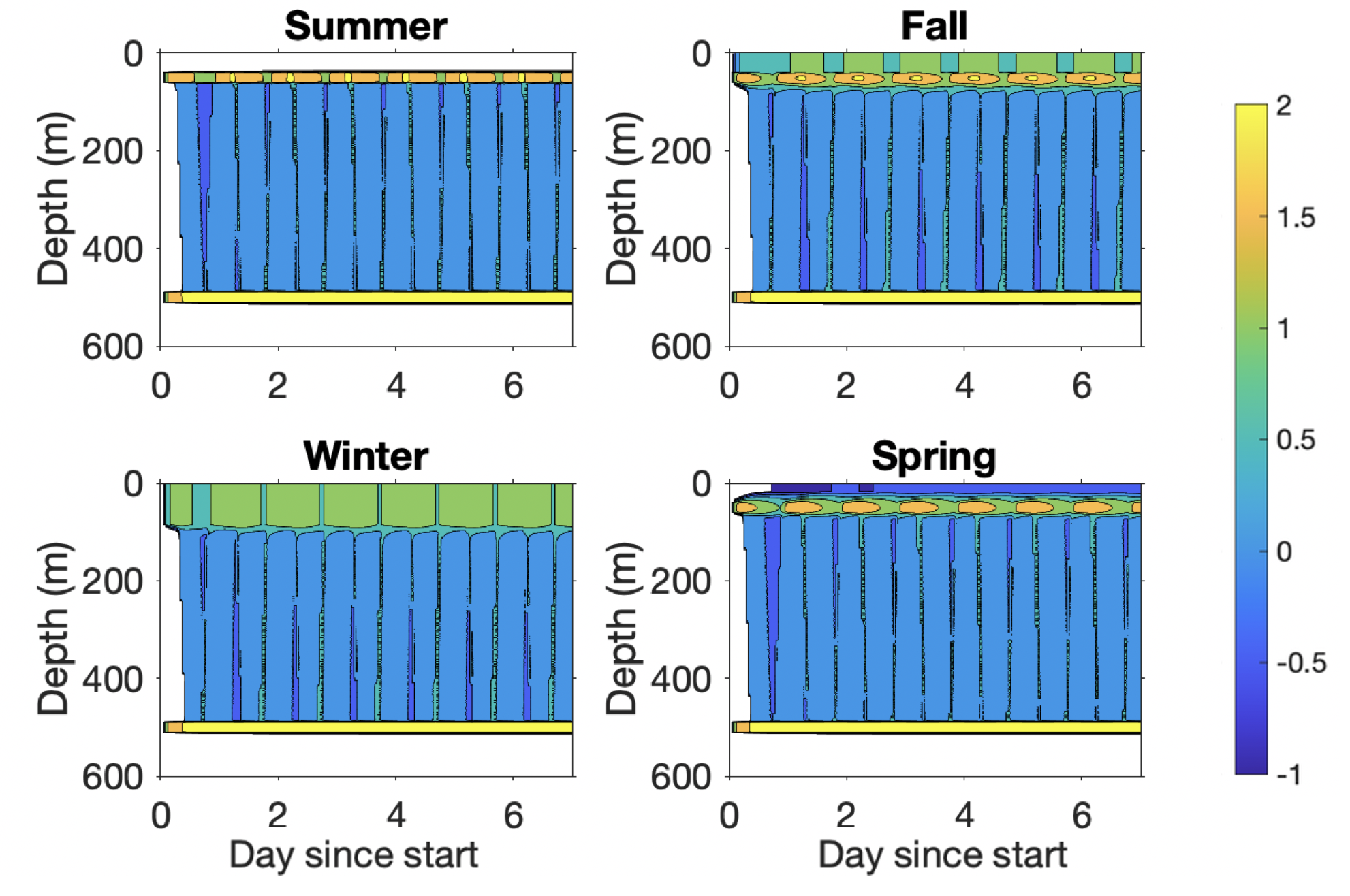


D)

C)

B)

A)

**Supplemental Figure S4. Temporal evolution of eDNA concentration vertical profiles in a representative simulation of each season.** Vertical profiles of log_10_(eDNA concentration) in the first seven days of the 90-day seasonal simulations are shown. Decay rate constant, vertical diffusivity profile, and animal migration times (as determined by sunrise and sunset) vary by season. Other parameters are held constant among the simulations. Panels A, B, C, and D show simulations for summer, fall, winter, and spring, respectively.


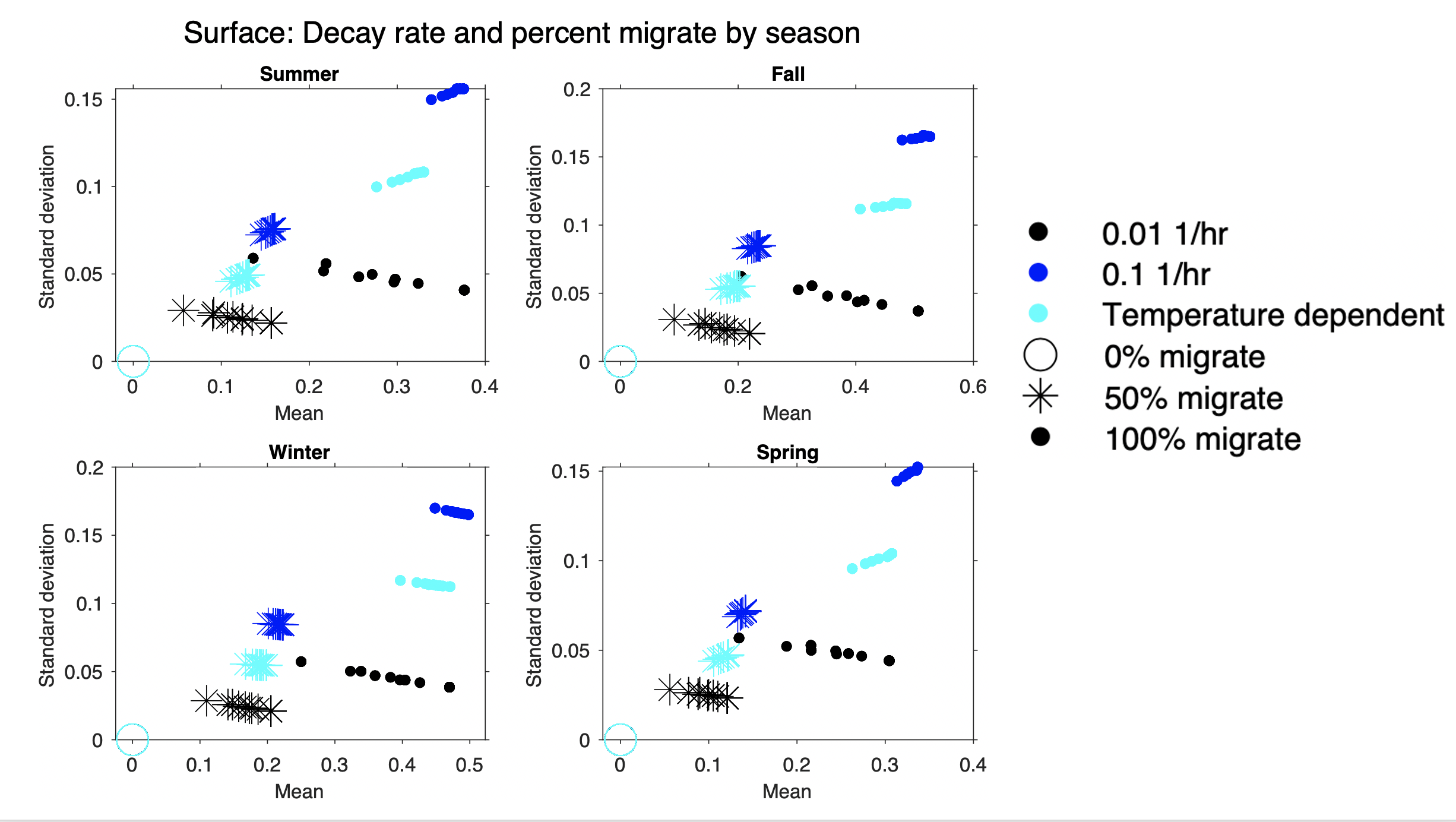

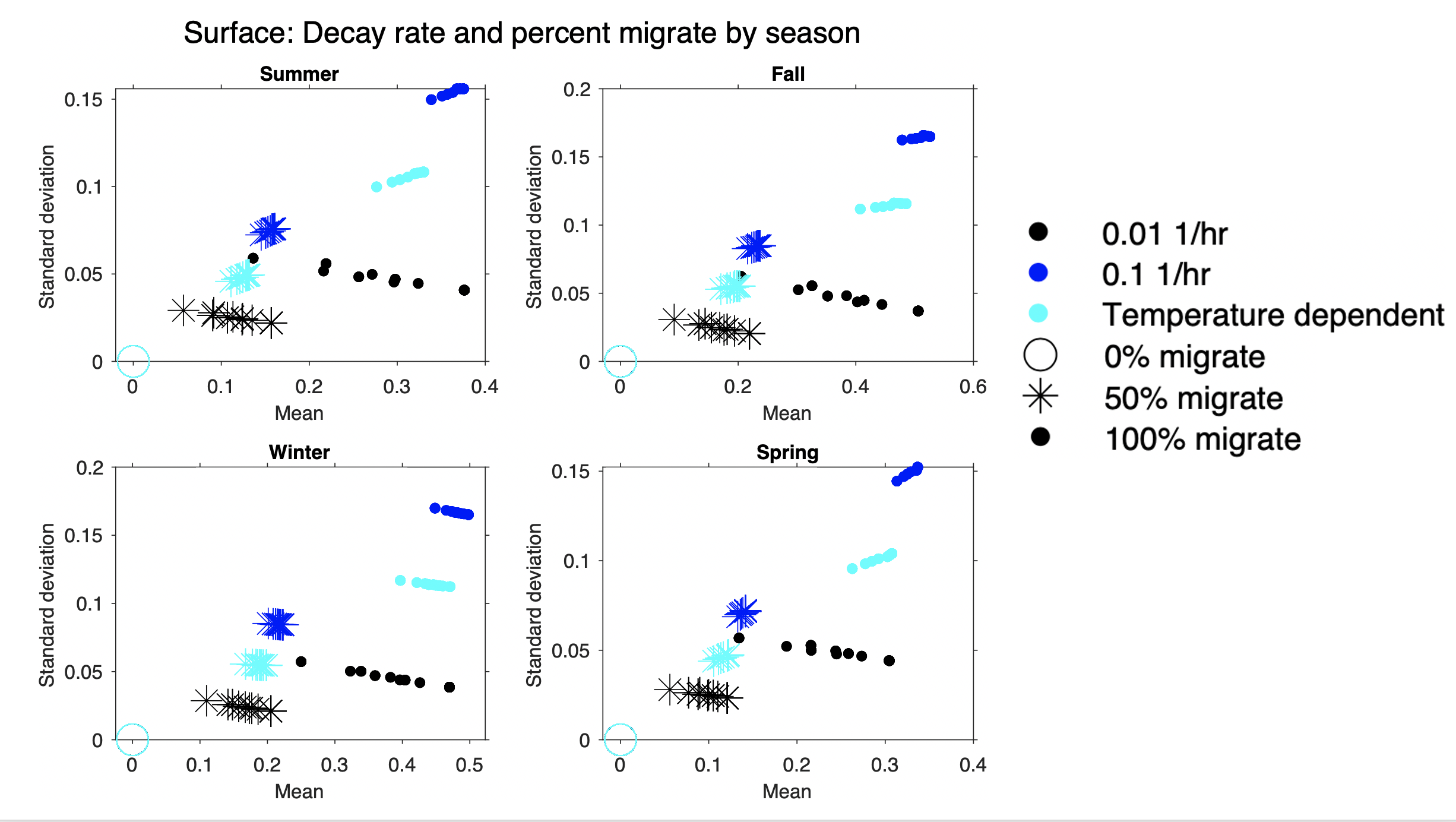


C)

D)

A)

B)


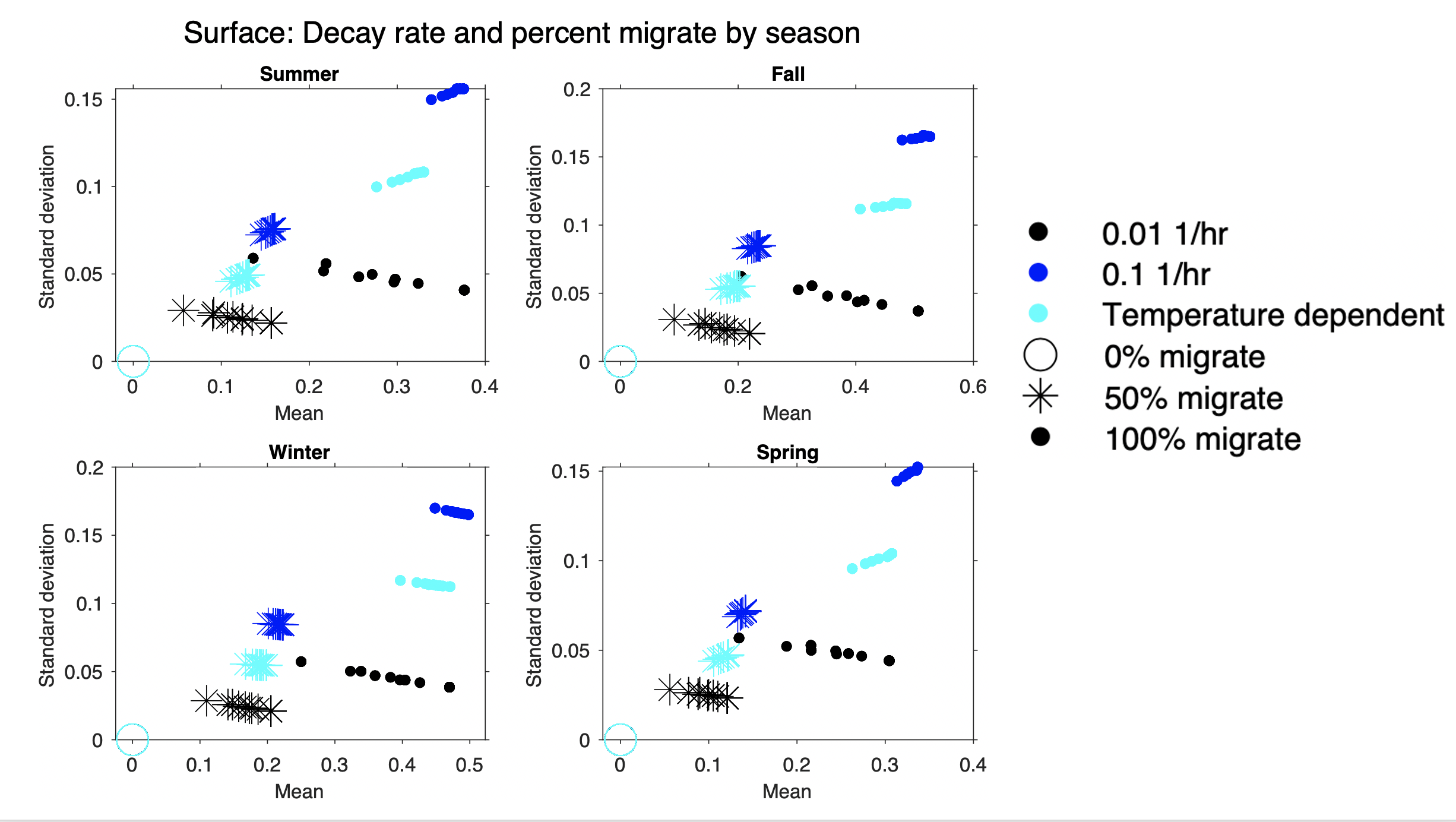


**Supplemental Figure S5. Proportion of eDNA found in the surface layer by season, decay rate constant, and percent of individuals that migrate.** The x- and y-axes represent the mean and standard deviation of the proportion of eDNA found in the surface layer (0-100 m) over the 90-day simulations, respectively. Each marker represents a different simulation in the sensitivity analysis. Colors of the markers correspond to the modeled decay rate constant. Shapes correspond to the percent of individuals that migrate. Panels A, B, C, and D show simulations for summer, fall, winter, and spring, respectively.

A)


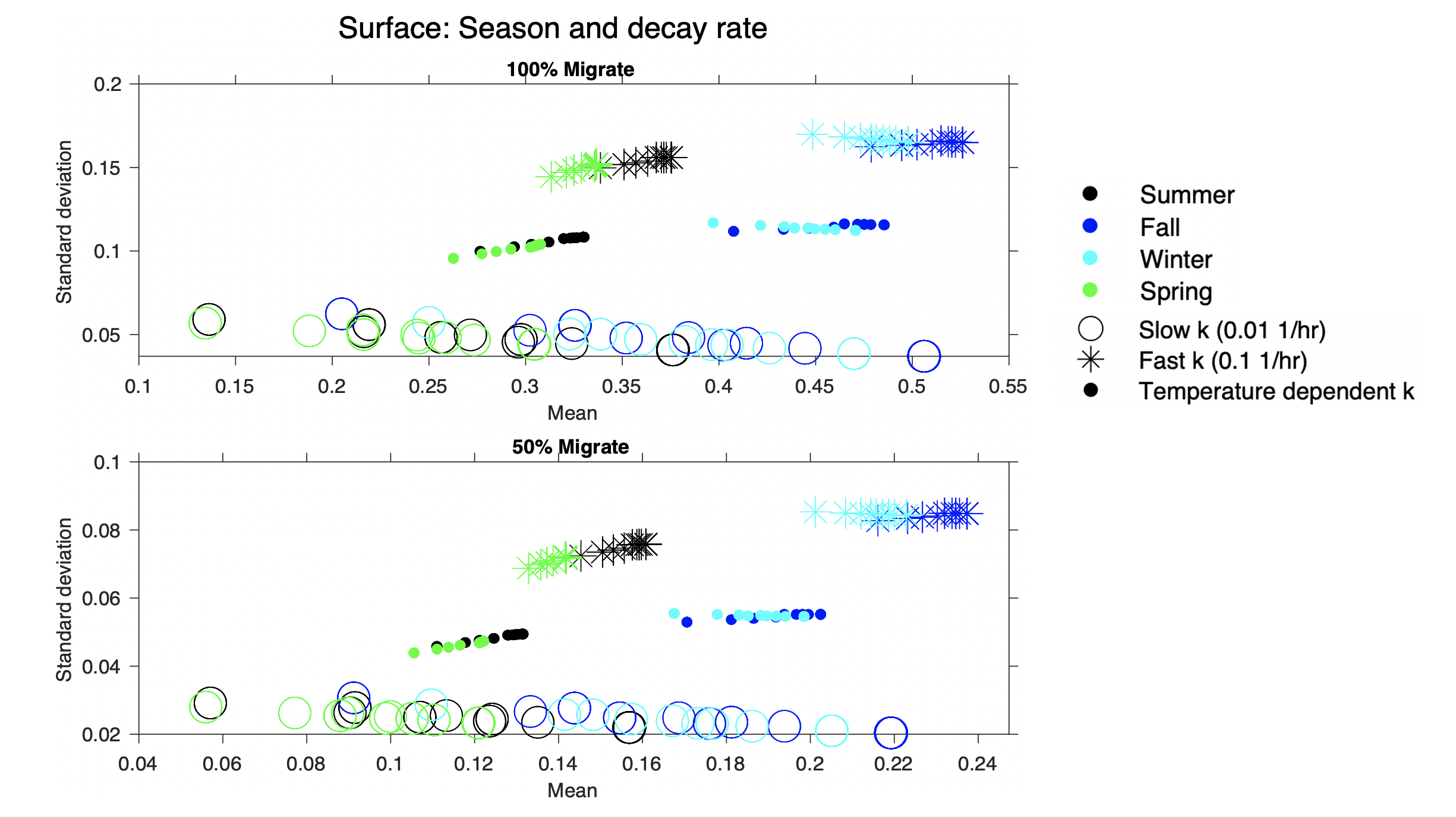


B)

**Supplemental Figure S6. Proportion of eDNA found in surface layer by season, decay rate constant for 50% or 100% of individuals migrating.** The x- and y-axes represent the mean and standard deviation of the proportion of eDNA found in the surface layer (0-100 m) over the 90-day simulation, respectively. Each marker represents a different simulation in the sensitivity analysis. Colors of the markers correspond to the season of the simulation. Shapes correspond to the modeled decay rate constant. Panel A shows simulations where 100% of the individuals migrate and Panel B shows simulations where 50% of the individuals migrate.

A)

B)

C)

**Supplemental Figure S7.** Ratio of average eDNA concentration in surface to deep layers as a function of percent of individuals that migrate in different seasons. Panel A shows regular seasons (same as Figure 6 in main text). Panel B shows simulation result with the physical forcings corresponding to the season but with the day length fixed at the summer value. Note that the day length determines sunrise and sunset and thus migration times. Panel C shows results of the simulations forced by summer physical forcing but with the sunrise and sunset corresponding to each season.

**References**

Holte, J., Talley, L. D., Gilson, J. & Roemmich, D., 2017: An argo mixed layer climatology and database. Geophys. Res. Lett. 44, 5618–5626, doi: https://doi.org/10.1002/2017GL073426

Stokes, G.G., 1851: On the effect of the internal friction of fluids on the motion of pendulums. *Trans. Camb. Phil. Soc.,* 9, 8-106.

Wilkin, J., et al., 2018: A coastal ocean forecast system for the U.S. Mid-Atlantic Bight and Gulf of Maine. In "*New Frontiers in Operational Oceanography*", E. Chassignet, A. Pascual, J. Tintoré, and J. Verron, Eds., GODAE OceanView, 593-624, doi:10.17125/gov2018.ch21.
